# Gastrointestinal quality of life in children born with gastroschisis

**DOI:** 10.1007/s00383-024-05909-4

**Published:** 2024-12-10

**Authors:** Matilda Bräutigam, Michaela Dellenmark-Blom, Kate Abrahamsson, Cathrine Gatzinsky, Vladimir Gatzinsky

**Affiliations:** Department of Pediatric Surgery, Queen Silvia Children’s Hospital, Sahlgrenska University, Hospital, Institute of Clinical Sciences, Sahlgrenska Academy, Gothenburg University, 416 85 Gothenburg, Sweden

**Keywords:** Gastroschisis, Quality of life, Gastrointestinal quality of life, Silo, PedsQL gastrointestinal symptom module

## Abstract

**Purpose:**

The aim was to determine gastrointestinal (GI)-related QoL in children born with gastroschisis (GS).

**Methods:**

Totally, 58/83 families of children (aged 2–18 years) operated for GS at a tertiary pediatric surgical center accepted participation. Children aged 5–18 and one parent (child aged 2–18) completed the Swedish version of the PedsQL™ gastrointestinal symptoms module, evaluating GI-related QoL with 14 different GI-specific scales, norm values for Hirschsprung’s disease (HD), esophageal atresia (EA), and functional constipation (FC) that were used for comparison.

**Results:**

Children with GS had significantly lower parent-reported scores on “Gas and bloating” compared with children with EA (77.0 vs 85.5, *p* = 0.039). In the child report and in the parent report, scores on several GI scales were like those of children with EA. Parents of children with GS had higher scores for 8/14 scales compared to HD and higher scores for 12/14 scales compared to FC. Clinical GS-specific factors for worse GI-QoL were identified, including “Days in ventilator” and “Days with Silo and Patch”.

**Conclusions:**

GS has an impact on GI-related QoL, comparable to that in EA, but not to HD or FC. The GS-specific factors of worse QoL show the importance regarding a GS follow-up program including considering clinical factors.

**Supplementary Information:**

The online version contains supplementary material available at 10.1007/s00383-024-05909-4.

## Introduction

Gastroschisis (GS) is a congenital birth defect where the intestines herniate through the abdominal wall on the right side of the umbilical cord. The incidence of GS differs worldwide. For example, American studies show an incidence of 4–5 per 10,000 live births, while European studies show 2.74 per 10,000 live births. In Sweden, a recent study showed that the birth incidence has been quite stable with 1.52 per 10,000 live births for the last 20 years [1]. The mortality in Sweden was 4.4% during 1997–2016 [1], while countries in a low-resource setting might have a mortality as high as 100% [2]. Although syndromes are rare, gastroschisis can be associated with other congenital anomalies such as cardiovascular or musculoskeletal, but gastrointestinal (GI) being the most common [1, 3]. GS is usually divided into two groups, simplex and complex, with the latter consisting of atresia, perforation, necrosis or stenosis [4].

As the survival rates have increased, focus has shifted from mortality towards long-term morbidity, However, knowledge of the child’s quality of life (QoL) is still sparse, which could help guide the healthcare system to better understand healthcare needs that are important for the child and thereby adjust their care [5]. Some studies of GS have shown a QoL comparable to the healthy population, while others report a poorer QoL [6–8]. Several studies mention GI problems [9, 10], but only one evaluated the number of GI symptoms and their effect on QoL [7]. QoL has been identified by parents, patients with gastroschisis and clinicians as one out of eight core outcomes set for GS [11]. Thus far, no study has compared the level of QoL in GS against other congenital malformations and if clinical factors affect the QoL.

The first study aim was to determine GI-related QoL in children born with GS using the Swedish version of the PedsQL gastrointestinal symptoms module (GSM) [12–14], a validated GI-QoL questionnaire with separate child and parent reports, compared to the GI-related QoL in children born with Hirschsprung’s disease (HD), esophageal atresia (EA), and functional constipation (FC). The other aim of the study was to determine if clinical GS-specific factors could be associated with a worse QoL, hypothesizing that a complicated neonatal period for children with GS could affect the long-term QoL outcomes.

## Materials and methods

### Patient selection and data collection

This is a cross-sectional study of all children (aged 2 to 18 years) born with GS in 2004–2021 and operated at a tertiary pediatric surgical center identified through surgical records. There were no exclusion criteria. The families were invited by phone by a research nurse. Written information about the study, informed consent, and the GSM (paper pencil version) were sent to the study participants by post. For children aged 5–7 years, the questionnaire was interview based and an interview was scheduled with the research nurse.

### The PedsQL™ gastrointestinal symptoms module

**Fig. 1:**
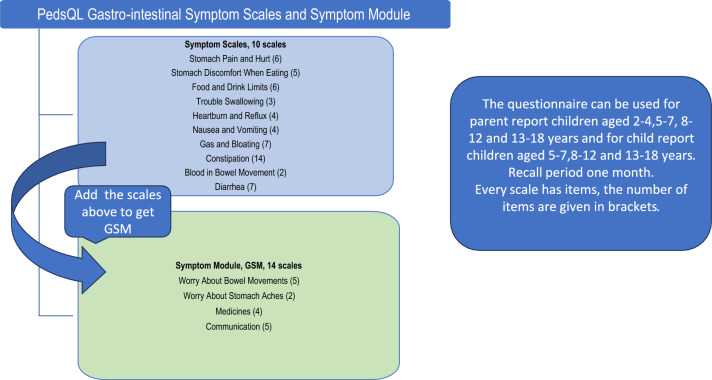
Description of the PedsQL gastrointestinal symptom scales and symptom module. The symptom scale consists of 10 scales and symptom module of 14 scales. The number of items in each scale is given in brackets

The PedsQL™ gastrointestinal symptoms module (GSM, 74 items) measures gastrointestinal-related health-related quality of life (HRQOL) in pediatric patients, but also enables assessment of GI-specific symptom scales and a GSM total score as presented in Fig. 1. GSM covers items from stomach pain, food issues, and bowel problems to worries. GSM can be used for children ages 2 to 4 years (parent-proxy reports) and ages 5 to 7 years, 8 to 12 years, and 13 to 18 years (child report and parent reports). Each item has a maximum score of 100 for excellent quality of life, while low scores reflect many gastrointestinal symptoms and low quality of life. The questions are the same for all age groups, but adjustments have been made in the language to fit every age group and school or day care activities. For children aged 5–7, the questionnaire is interview-based and uses a 3-point response scale. Children aged 8–12 and 13–18 years answer the questions themselves and a 5-point response scale is used. For parent reports with children aged 2–4, 5–7, 8–12, and 13–18 years old, a 5-point response scale is used [13, 14].

### Factors associated with QoL

The study started with a set of clinical factors covering a wide area of aspects for example mothers age, prenatally known diagnosis, silo, bowel resection and nutrition when the child went home. Based on the literature and discussion in the author team, the factors chosen were those that we hypothesized would correlate with QoL and that were previous known risk factors for complications in children born with gastroschisis [15, 16].

#### Comparison group

The results from a previous study done by our research group children with EA, HD and FC answering the GSM were used for comparison with GS in this study [17].

### Statistical analysis

For statistical analysis, SAS 9.4 (SAS Institute Inc., Cary, NC, USA) was used. Descriptive statistics were used to describe patient characteristics. The response options of the PedsQL GI scales were transformed to a linear score scale with 0 denoting worst and 100 best GI-QoL. P values < 0.05 were considered significant. For categorical variables, *n* (%) is presented and for continuous variables, and mean (SD) is presented. Pearson correlation coefficient was used to measure correlations between the scale results and the clinical parameters, ± 1 was considered perfect correlation, ± 0.50 to ± 1 a high degree, ± 0.30 to ± 0.49 moderate and below ± 0.29 low degree of correlation. The Mann–Whitney *U* test was used to compare between groups for continuous variables related to PedsQL and demographics. Comparison between gastroschisis and the other different patient groups, i.e., EA, HD, and FC were calculated with *T*-test. As reference, results from two studies on the Swedish GSM were used [12, 17].

This study was performed in line with the principles of the Declaration of Helsinki. Approval was granted by the Swedish Ethical Review Authority (2022-01838-01).

## Results

### Patients

In total, 84 patients, aged 2–18 years, were identified between December 2022 and June 2023, of which 71 could be reached. Fifty-eight (82%) agreed to participate in the study. Three of the initial patients (*n* = 84) had complex GS, of which one participated in the study. Table 1 shows the demographic characteristics of children with GS. Out of 58 children, 10 were treated with patch, 10 with silo out of whom 4 were followed by a patch.

**Table 1:** Demographics for recruited GS patients

Variable	All (*n* = 58)	Gestational age (in weeks), *n* = 58	
Age (*y*), mean (SD)	10.5 (4.5)	31	2 (3.4%)
Participants in each age group		32	6 (10.3%)
2–4 years	7 (12.1%)	33	4 (6.9%)
5–7 years	16 (27.6%)	34	9 (15.5%)
8–12 years	18 (31.0%)	35	13 (22.4%)
13–18 years	17 (29.3%)	36	22 (37.9%)
Gender		37	2 (3.4%)
Male	18 (31.0%)	Birth weight (kg)	2.28 (0.50)
Female	40 (69.0%)		
Mothers age at birth of child (y)	26.1 (5.2)		

### Factors associated with QoL in GS

For children born with GS, the GSM total score was 84.1 (SD, 12.3) for the child report and 87.5 (SD, 11.7) for the parent report. The following factors served for investigation of hypothesized risk factors for worsened GI-QoL: “Younger child age”, “Days with silo”, “Days with silo and patch”, “Days in ventilator”, “Age for total enteral nutrition”, “Days on enteral feeds before full enteral nutrition” referring to how many days the child had partial oral nutrition, “Wound infection”, “Ischemic bowel” referring to ischemic bowel at birth and “Number of anesthetics”.

According to the parent reports, the age group 5–7 scored lower than 8–12-year-olds on five scales and in the GSM total score and GI symptoms total score. In addition, the 5–7-year-olds also scored worse on the “Constipation” (*p* = 0.04) and “Communication” (*p* = 0.02) scales compared to 13–18-year-olds. The score for the “Trouble swallowing” scale was significantly higher for 8–12-year-olds (100) compared to 2–4-year-olds (96.6; *p* = 0.02), (Supplemental table S4). Significant differences in “Stomach pain and hurt” were found between boys (mean score 88.5; SD, 12.2) and girls (mean score 72.0; SD, 23.9) in the child report (*p* = 0.019), but not for any other domain (Supplemental table S1) or in the parent report (Supplemental table S2). In the child report, children aged 5–7 years scored higher on the “Nausea and Vomiting” compared to age group 8–12 (*p* = 0.02) but scored lower on “Medicines” (*p* = 0.01) (S3). No other differences were seen between the different age groups. The one factor which seemed to be most often negatively associated with GI-QoL was “Days on ventilator”, which affected five scales (“Food and Drink Limits”, “Trouble Swallowing”, “Heartburn and Reflux”, “Nausea and Vomiting”, “Gas and Bloating”) as well as the GSM total score and GI symptom total score (parent and child reports). Other factors such as “Days with silo” and “Days with silo and patch” were also negatively associated with “Stomach pain and hurt” and “Stomach Discomfort When Eating” (child report) and strongly with “Food and Drink Limits”, “Nausea and Vomiting” and “Worry about Stomach Aches” (parent report; Supplemental table S5***)***. The absence of the factors “Wound infection” and “Ischemic bowel” were correlated to a better outcome in scales “Trouble Swallowing” and “Worry About Going Poop” for both the parent and child reports (Supplemental Table S6).

### QoL in GS compared to other conditions

Children with GS (*n* = 58) had significantly lower parent-reported scores on the “Gas and bloating” scale compared to the parent report for children with EA (*n* = 32; mean score 77.0 vs 85.5, *p* = 0.039, Supplemental table S7). However, the scores for several GI scales were generally similar on the child report (Supplemental table S8) and parent report for children with EA. Compared to HD, parents of children with GS rated higher on the GI module total score (71.9 vs 87.5; *p* < 0.001) as well as had significantly higher scores in 8/14 scales. On the child report, children with FC scored significantly worse in 5/14 scales compared to GS, which was 12/14 for the parent report. In the child report, children with GS had significantly higher GI module total scores (mean score, 84.6) than those with FC (mean score, 76.2; *p* = 0.01), but not EA and HD. In the parent report, the GI module total score was higher for children with GS than HD (87.5 vs 72.6; *p* < 0.001) and GS and FC (87.5 vs 75.3; *p* < 0.001), but not EA (87.5 vs 75.3; *p* < 0.0001) (Supplemental table S7).

## Discussion

This is, to our knowledge, the largest study to evaluate QoL in children born with GS, including exploring associated clinical factors and GI-QoL, reporting the view of the children and comparing the results to other GI disorders [8, 18, 19]. In fact, most previous studies are parent reported and small [6, 7]. A study by De Bie et al. found that QoL did not differ from healthy controls [7], whereas Arnold et al. [20] found using the same questionnaire that the GI module total scores were similar between simplex and complex GS though the complex GS patients scored significantly lower on certain scales. As we only had one complex GS in our study, this comparison was not feasible. Interestingly, we only found a difference in the total score, when comparing answers in the parent report between 5 and 7 years compared to 8 and 12 years and no differences in the child report. The reason for the better score in the older group might be due to an adaptation and coping among the patients as they got further away from the initial trauma or an improvement in GI-related morbidity. In the child report, there were few differences comparing different age groups which is interesting since children have shown different perception of their QoL in different ages [21].

In the child report, girls had statistically worse scores for “Stomach Pain and Hurt” than boys. The reason for this is unknown though it has been shown that girls seem to have more problems with abdominal pain than boys [22]. When it comes to our findings that children in age group 5–7 scored lower than children in age group 8–12 in “Medicines”, one might speculate that the lower score for the younger group is caused by a reluctance to take medicine. The 8–12 age group might have scored worse on “Nausea and vomiting” than children aged 5–7 years due to children at this age being increasingly able to express themselves.

An analysis of the parent report showed that there was a clear difference between several scales comparing 5–7- and 8–12-year-olds, with a worse outcome in the younger group. It has been described that children raise their parents’ concerns more when they are young compared to when they are older [23]. We found that “Age for total oral nutrition” correlated moderately with “Trouble swallowing”, “Heartburn and Reflux” and “Diarrhea” in the child report but not in the parent report. Kamity et al. [24] who investigated feeding problems among preterm infants stated that the underlying causes are multifactorial, also mentioning GS as a condition linked to dysphagia. Oral phase disruption which affects oral stimulation causing problems with oral intake might cause dysphagia but on the other hand trouble swallowing for whatever cause and gastroesophageal reflux might aggravate or even cause delayed oral intake. Gastroesophageal reflux in neonates has been shown to cause problems with feeding, dysphagia, and even oral aversion [25]. The prevalence of gastroesophageal reflux in GS has been shown to range between 16 and 30%, with a decline with age [7, 20, 26].

We found that “Days with silo” and “Days with silo and patch” correlated negatively to scales in both the child and parent reports. It has previously been shown that the earlier a closure of the silo is done, the sooner the start of enteral feeds, shorter time to full enteral feeds, time in ventilator, and avoidance of infections [27–29]. The context of this correlation is not known. It might be an expression of a more severe disease with more inflammatory bowel or there might be the silo itself that for some reason causes those problems. However, Hawkins et al. showed that children with immediate closure and children with silo < 5 days did not differ in outcome during their first hospital admission [28]. “Days in ventilator” was the single factor with most correlations of all clinical factors to scales in both the child and parent reports. There were evidently many upper GI problems in this group and the GSM total score, and GI symptoms total score correlated for both the child and parent. Children that have been placed on a ventilator for a long time have been found to have different concerns regarding swallowing leading to feeding problems [30]. Several studies have been done on the influence of anesthesia, with differing results. Some studies have found that anesthesia affects the brain, resulting in neurotoxicity while this could not be confirmed by others [31–33]. Although we did not investigate any of those aspects, we found that more “Days in ventilator” was associated with a worse QoL than those having less.

Wound infections are mostly related to delayed closure of the abdomen and silo and has been described in up to 34% [7, 20]. In the present study, 10 children were treated with silo of which 4 were treated with silo followed by patch. We found that “Wound infection” correlated moderately too high in the child report (“Trouble swallowing”, “Constipation”, “Worry about going poop”) and moderately in the parent report (“Trouble swallowing”, “Worry about going poop”, “Communication”). This connection has not previously been described. It has been shown that wound infections negatively impact physical health, increase the length of stay at hospital, and increase morbidity [34]. Children with wound infections might, because of the infection, have a slower start in the development of oral feeding which causes future problems with swallowing, given the swallowing reflex did not have the opportunity to develop as it should have [24].

Not surprisingly, children with EA showed lower scores in the scales “Trouble swallowing” and “Heartburn and reflux” than children with GS in the parent report. It is well known that dysphagia and gastroesophageal reflux are some of the main problems among children with EA [35]. This might explain why children with EA had a lower score when it came to “Medication”, being a patient group which often requires medicine for both gastroesophageal and respiratory problems. Interestingly, patients with GS showed lower scores when it came to “Gas and bloating” (parent report) and “Blood in poop” (child report). It might not be too surprising though since GI symptoms are quite common in this patient group [36]. The GSM total score and GI symptoms total scores were the same for EA and GS, both in the parent and child reports. Since we know that there are differences between these two patient groups, this might show that a symptom-specific questionnaire might not be the best for describing QoL differences between different malformations.

Children with HD are known to have a worse QoL compared to healthy controls [37, 38]. We found that children with GS and HD in the child report did not differ between the groups when it came to GSM total score or GI symptoms total score. The fact that patients with HD scored lower in the scale “Diarrhea” is not unexpected since this is usually not a problem in the GS group, but is well known for being one of the problems associated with outcome of patients surgically treated for HD [39]. In the parent report, HD had a worse outcome in almost every GI scale, reflecting the problems which patients with HD often describe. This finding is encouraging for children with GS, however as other studies indicate a quite high number of gastrointestinal symptoms [9, 36] our low prevalence of complex GS cases should be considered. Comparing GS and FC showed that FC had a worse QoL than GS in both the child and parent reports. It is well known that children with FC often score worse than healthy children and even worse than children with organic GI disorders QoL [40].

## Study limitations

With a higher number of patients with GS, a multivariable regression analysis of the most contributing factors to QoL could have been performed. We did not have a healthy reference group as comparison for GI-QoL, which might have been interesting but on the other hand, this was not the aim of the study. Since non-participants did not give us informed consent to participate in the study, we could not review their medical records and analyze potential differences between participants and non-participant. This could be understood as a study limitation. However, compared to other QoL studies in gastroschisis with participation rates of 24% [6], 33% [41] and 68% [19], our participation rate was acceptable. Although we have not changed the operation method during the 16 years that the study represents, even small differences in operation technique and differences in anesthesia might affect the outcome. Due to different changes in the PN during the reported period, we could not do any reliable analyses of which impact this might have had on the QoL. Another aspect to explore is the parents view on major complications. Those aspects could be something for further studies to investigate.

## Conclusion

The study demonstrates that the level of gastrointestinal QoL in children with GS in parts is similar to that in children with EA and HD, both groups previously known to have negatively affected QoL. Furthermore, children with GS have several clinical risk factors that might affect their GI-QoL of which “Days in ventilator” seems to have the greatest impact. The clinical factors identified could be used as a tool to identify children at risk and follow them closely in the healthcare system. Pediatric surgeons need to further investigate the needs of follow-up in children with GS, QoL included. A diagnosis-specific questionnaire for children with GS could be a way to better explore their QoL, avoiding the risk of missing specific areas of the patients’ interest.

## Supplementary Information

Below is the link to the electronic supplementary material.

Supplementary file1 (DOCX 57 kb)

## Data Availability

No datasets were generated or analysed during the current study.
