# Supplementary material for: Gastrointestinal quality of life in children born with gastroschisis
Source: Pediatr Surg Int. 2024 Dec 10;41(1):24. doi: 10.1007/s00383-024-05909-4 (PMC11632061; doi:10.1007/s00383-024-05909-4)
Supplement: Supplementary file 1 — Supplementary file1 (DOCX 57 kb) [file 383_2024_5909_MOESM1_ESM.docx]

**Supplemental**

Abbreviations used:

GI-gastrointestinal

QoL-quality of life

GSM- PedsQL Gastrointestinal Symptoms Module

GI symptom score - PedsQL Gastrointestinal Symptoms Scales

Table S1. PedsQL gastrointestinal symptoms scales and worry scales for child report total and by gender**.**

| **Variable-Scale** | **Total (n=48)** | **Male (n=13)** | **Female (n=35)** | **p-value** |
| --- | --- | --- | --- | --- |
| Stomach Pain and Hurt | 76.5 (22.5) | 88.5 (12.2) | 72.0 (23.9) | **0.019** |
| Stomach Discomfort When Eating | 87.8 (16.2) | 92.3 (8.1) | 86.1 (18.2) | 0.44 |
| Food and Drink Limits | 86.1 (25.0) | 89.4 (19.5) | 84.9 (27.0) | 0.93 |
| Trouble Swallowing | 93.1 (14.1) | 93.6 (14.1) | 92.9 (14.3) | 0.98 |
| Heart Burn and Reflux | 86.1 (15.8) | 88.9 (13.3) | 85.0 (16.6) | 0.48 |
| Nausea and Vomiting | 93.9 (12.8) | 94.2 (9.4) | 93.8 (14.0) | 0.55 |
| Gas and Bloating | 75.0 (24.6) | 79.9 (21.1) | 73.1 (25.9) | 0.55 |
| Constipation | 77.8 (19.3) | 81.0 (18.3) | 76.7 (19.7) | 0.53 |
| Blood in Poop | 95.1 (14.3) | 98.1 (6.9) | 93.9 (16.1) | 0.42 |
| Diarrhea | 88.5 (16.3) | 92.1 (12.6) | 87.2 (17.5) | 0.32 |
| Worry About Going Poop | 91.5 (17.2) | 93.2 (19.5) | 90.9 (16.7) | 0.18 |
| Worry About Stomach Aches | 84.1 (27.5) | 85.4 (22.5) | 83.6 (29.5) | 0.94 |
| Medicines | 90.9 (19.2) | 89.1 (23.3) | 91.7 (17.6) | 0.83 |
| Communication | 81.7 (28.1) | 88.3 (21.6) | 79.0 (30.4) | 0.15 |
| GSM total score | 84.1 (12.3) | 87.9 (11.4) | 82.7 (12.5) | 0.17 |
| GI symptoms total score | 83.4 (12.9) | 87.6 (11.4) | 81.8 (13.2) | 0.14 |
| For continuous variables Mean (SD) is presented. For comparison between groups the Mann-Whitney U-test was used for continuous variables. GSM total score- 74 items, GI symptoms total score- 58 items. The response options of the PedsQL Gastro-intestinal scales were transformed to a linear score scale with 0 denoting worst and 100 best quality of life. p< 0.05 marked in bold text, considered significant. | | | | |

Table S2. PedsQL gastrointestinal symptoms scales and worry scales for parent report total and by gender.

| **Variable-Scale** | **Total (n=57)** | **Male (n=18)** | **Female (n=39)** | **p-value** |
| --- | --- | --- | --- | --- |
| Stomach Pain and Hurt | 81.4 (19.0) | 84.7 (15.8) | 79.9 (20.3) | 0.59 |
| Stomach Discomfort When Eating | 87.5 (18.2) | 90.6 (11.4) | 86.0 (20.6) | 0.64 |
| Food and Drink Limits | 90.9 (19.6) | 87.5 (26.0) | 92.5 (16.0) | 0.62 |
| Trouble Swallowing | 98.5 (3.9) | 97.2 (5.7) | 99.1 (2.6) | 0.20 |
| Heart Burn and Reflux | 92.7 (10.5) | 92.7 (9.6) | 92.6 (11.0) | 0.93 |
| Nausea and Vomiting | 95.1 (10.7) | 95.1 (10.4) | 95.0 (11.0) | 0.86 |
| Gas and Bloating | 77.0 (23.4) | 76.2 (21.9) | 77.4 (24.3) | 0.77 |
| Constipation | 82.3 (19.1) | 84.8 (16.8) | 81.1 (20.1) | 0.42 |
| Blood in Poop | 95.6 (10.4) | 97.2 (6.9) | 94.9 (11.7) | 0.66 |
| Diarrhea | 92.3 (12.4) | 93.3 (10.2) | 91.8 (13.3) | 0.92 |
| Worry About Going Poop | 94.6 (10.8) | 95.3 (13.2) | 94.4 (9.7) | 0.40 |
| Worry About Stomach Aches | 88.8 (21.0) | 93.1 (16.2) | 86.9 (22.8) | 0.34 |
| Medicines | 95.3 (13.0) | 97.6 (6.5) | 94.3 (15.2) | 0.60 |
| Communication | 79.6 (27.0) | 76.7 (32.9) | 81.1 (24.2) | 0.99 |
| GSM total score | 87.5 (11.7) | 88.3 (11.8) | 87.2 (11.8) | 0.56 |
| GI symptoms total score | 87.0 (12.7) | 87.9 (12.0) | 86.6 (13.1) | 0.69 |
| For continuous variables Mean (SD) is presented. For comparison between groups the Mann-Whitney U-test was used for continuous variables. GSM total score- 74 items, GI symptoms total score- 58 items. The response options of the PedsQL Gastro-intestinal scales were transformed to a linear score scale with 0 denoting worst and 100 best quality of life. p< 0.05 marked in bold text, considered significant. | | | | |

S3. PedsQL gastrointestinal symptoms scales and worry scales means (standard deviation) for child self-report by age groups.

|  | | | | **Test between age-groups p-value** | | |
| --- | --- | --- | --- | --- | --- | --- |
| **Variable** | **5-7 years (n=15)** | **8-12 years (n=16)** | **13-18 years (n=17)** | **5-7 vs 8-12** | **5-7 vs 13-18** | **8-12 vs 13-18** |
| Stomach Pain and Hurt (Child) | 72.8 (27.7) | 80.2 (16.3) | 76.2 (23.2) | 0.65 | 0.83 | 0.88 |
| Stomach Discomfort When Eating (Child) | 90.7 (8.8) | 89.4 (13.9) | 83.8 (22.3) | 0.85 | 0.75 | 0.63 |
| Food and Drink Limits (Child) | 85.0 (30.2) | 83.3 (29.9) | 89.7 (13.5) | 0.50 | 0.28 | 0.72 |
| Trouble Swallowing (Child) | 92.2 (18.8) | 93.8 (10.8) | 93.1 (12.9) | 0.64 | 0.52 | 0.93 |
| Heart Burn and Reflux (Child) | 82.5 (17.6) | 89.8 (14.9) | 85.7 (14.9) | 0.20 | 0.62 | 0.25 |
| Nausea and Vomiting (Child) | 99.1 (3.3) | 93.0 (10.7) | 90.4 (17.8) | 0.017 | 0.10 | 0.58 |
| Gas and Bloating (Child) | 81.9 (16.4) | 77.1 (24.6) | 66.8 (29.3) | 0.92 | 0.20 | 0.34 |
| Constipation (Child) | 73.2 (20.6) | 78.8 (17.5) | 81.0 (20.0) | 0.55 | 0.33 | 0.53 |
| Blood in Poop (Child) | 95.0 (10.4) | 95.3 (18.8) | 94.9 (13.3) | 0.32 | 0.89 | 0.39 |
| Diarrhea (Child) | 89.0 (12.6) | 88.4 (19.5) | 88.2 (16.9) | 0.58 | 0.87 | 0.71 |
| Worry About Going Poop (Child) | 96.0 (7.9) | 87.9 (22.7) | 91.2 (17.1) | 0.33 | 0.43 | 0.79 |
| Worry About Stomach Aches (Child) | 86.5 (30.0) | 83.9 (28.8) | 82.4 (26.2) | 0.59 | 0.31 | 0.69 |
| Medicines (Child) | 86.5 (17.3) | 100.0 (0.0) | 87.5 (25.9) | 0.0099 | 0.59 | 0.017 |
| Communication (Child) | 85.8 (16.8) | 79.6 (36.6) | 80.3 (29.1) | 0.71 | 0.98 | 0.76 |
| GI module total score (Child) | 84.1 (8.7) | 84.9 (12.9) | 83.4 (15.0) | 0.49 | 1.00 | 1.00 |
| GI symptoms total score (Child) | 82.9 (10.0) | 84.4 (12.9) | 82.9 (15.5) | 0.65 | 0.78 | 1.00 |
| For continuous variables Mean (SD) is presented. For pairwise comparison between groups the Mann-Whitney U-test was used for continuous variables.  Marked in yellow are the statistically significant p value (0,05) between groups. | | | | | | |

.

Table S 4. PedsQL gastrointestinal symptoms scales and worry scales for parents by age groups.

|  | | | | | **Test between age-groups, p-value** | | | | | |
| --- | --- | --- | --- | --- | --- | --- | --- | --- | --- | --- |
| **Variable** | **2-4 years (n=7)** | **5-7 years  (n=16)** | **8-12 years  (n=18)** | **13-18 years (n=16)** | **2-4 vs 5-7** | **2-4 vs 8-12** | **2-4 vs 13-18** | **5-7 vs 8-12** | **5-7 vs 13-18** | **8-12 vs 13-18** |
| Stomach Pain and Hurt (Parent) | 87.5 (13.4) | 73.4 (19.8) | 85.5 (16.4) | 82.0 (21.7) | 0.100 | 0.83 | 0.81 | 0.044 | 0.15 | 0.99 |
| Stomach Discomfort When Eating (Parent) | 90.7 (12.4) | 80.6 (18.0) | 93.9 (11.2) | 85.6 (24.5) | 0.19 | 0.56 | 0.80 | 0.0060 | 0.17 | 0.31 |
| Food and Drink Limits (Parent) | 92.3 (20.5) | 87.2 (26.7) | 93.8 (13.9) | 90.9 (17.7) | 0.29 | 0.49 | 0.39 | 0.54 | 0.82 | 0.69 |
| Trouble Swallowing (Parent) | 96.4 (6.6) | 98.4 (3.4) | 100.0 (0.0) | 97.9 (4.8) | 0.55 | 0.024 | 0.61 | 0.063 | 0.96 | 0.063 |
| Heart Burn and Reflux (Parent) | 93.8 (8.8) | 91.4 (10.7) | 95.1 (8.2) | 90.6 (13.3) | 0.65 | 0.45 | 0.72 | 0.23 | 1.00 | 0.23 |
| Nausea and Vomiting (Parent) | 94.6 (11.7) | 94.5 (10.9) | 96.9 (6.5) | 93.8 (14.3) | 0.90 | 0.75 | 0.97 | 0.71 | 0.98 | 0.80 |
| Gas and Bloating (Parent) | 79.1 (26.7) | 73.4 (19.1) | 81.3 (24.3) | 74.8 (26.2) | 0.40 | 0.92 | 0.92 | 0.14 | 0.82 | 0.61 |
| Constipation (Parent) | 76.8 (14.5) | 72.9 (23.3) | 89.5 (12.5) | 86.0 (19.2) | 1.00 | 0.093 | 0.12 | 0.012 | 0.038 | 0.71 |
| Blood in Poop (Parent) | 91.1 (18.7) | 93.0 (10.2) | 97.9 (6.4) | 97.7 (9.4) | 0.81 | 0.30 | 0.17 | 0.081 | 0.057 | 0.70 |
| Diarrhea (Parent) | 88.3 (9.4) | 92.4 (13.7) | 91.1 (14.8) | 95.3 (9.0) | 0.23 | 0.25 | 0.065 | 0.88 | 0.54 | 0.41 |
| Worry About Going Poop (Parent) | 97.9 (3.9) | 93.4 (9.8) | 95.0 (10.0) | 94.1 (14.6) | 0.38 | 1.00 | 1.00 | 0.33 | 0.34 | 0.93 |
| Worry About Stomach Aches (Parent) | 96.4 (6.1) | 91.4 (22.7) | 84.7 (22.5) | 87.5 (21.9) | 0.85 | 0.36 | 0.68 | 0.21 | 0.46 | 0.70 |
| Medicines (Parent) | 98.2 (4.7) | 93.8 (9.7) | 99.6 (1.5) | 91.0 (21.5) | 0.32 | 0.51 | 0.55 | 0.040 | 0.79 | 0.12 |
| Communication (Parent) | 68.6 (33.3) | 67.5 (28.9) | 87.1 (23.1) | 88.8 (21.6) | 0.89 | 0.19 | 0.076 | 0.039 | 0.015 | 0.51 |
| GI module total score (Parent) | 86.8 (11.6) | 83.0 (12.0) | 91.1 (8.9) | 88.4 (13.5) | 0.53 | 0.40 | 0.57 | 0.043 | 0.086 | 0.86 |
| GI symptoms total score (Parent) | 86.3 (12.3) | 82.3 (13.1) | 90.8 (9.8) | 87.7 (14.8) | 0.53 | 0.36 | 0.55 | 0.047 | 0.11 | 0.76 |
| For continuous variables Mean (SD) is presented. For pairwise comparison between groups the Mann-Whitney U-test was used for continuous variables.  Marked in yellow are the statistically significant p value (0,05) between groups. | | | | | | | | | | |

Table S5. Negative Pearson correlations for clinical factors and scales

|  | | **Parent report**  **clinical factors** | | | **Child report**  **clinical factors** | | | | | |
| --- | --- | --- | --- | --- | --- | --- | --- | --- | --- | --- |
| **Scale** | | **“Days in ventilator”** | **“Days with silo”** | **“Days with silo and patch”** | **“Days in ventilator”** | **“Days with silo”** | **“Days with silo and patch”** | **“Days on enteral feeds before full enteral nutrition”** | **“Age for total enteral nutrition”** | **“Number of anesthetics”** |
| **Stomach Pain and Hurt** | |  |  |  |  |  |  |  |  |  |
| Pearson Correlation Coefficients | | -0.20 | -0.34 | -0.94 | -0.22 | **-0.67** | -0.67 | 0.15 | 0.12 | 0.004 |
| p value | | 0.128 | 0.376 | 0.221 | 0.141 | **0.048** | 0.329 | 0.306 | 0.436 | 0.978 |
| Number of Observations | | 57 | 9 | 3 | 48 | 9 | 4 | 48 | 48 | 47 |
| **Stomach Discomfort When Eating** | |  |  |  |  |  |  |  |  |  |
| Pearson Correlation Coefficients | | -0.26 | -0.65 | -0.95 | **-0.36** | -0.60 | **-0.96** | 0.02 | -0.04 | -0.14 |
| p value | | 0.055 | 0.057 | 0.195 | **0.013** | 0.087 | **0.0442** | 0.897 | 0.809 | 0.351 |
| Number of Observations | | 57 | 9 | 3 | 48 | 9 | 4 | 48 | 48 | 47 |
| **Food and Drink Limits** | |  |  |  |  |  |  |  |  |  |
| Pearson Correlation Coefficients | | **-0.39** | **-0.67** | -0.99 | -0.06 | -0.37 | 0.49 | -0.17 | -0.16 | -0.02 |
| p value | | **0.003** | **0.047** | 0.068 | 0.666 | 0.326 | 0.510 | 0.242 | 0.273 | 0.868 |
| Number of Observations | | 57 | 9 | 3 | 48 | 9 | 4 | 48 | 48 | 47 |
| **Trouble Swallowing** | |  |  |  |  |  |  |  |  |  |
| Pearson Correlation Coefficients | | **-0.32** | 0.01 | 0.49 | -0.26 | -0.06 | 0.17 | **-0.47** | **-0.45** | **-0.34** |
| p value | | **0.017** | 0.980 | 0.672 | 0.076 | 0.876 | 0.831 | **0.001** | **0.002** | **0.020** |
| Number of Observations | | 57 | 9 | 3 | 48 | 9 | 4 | 48 | 48 | 47 |
| **Heart Burn and Reflux** | |  |  |  |  |  |  |  |  |  |
| Pearson Correlation Coefficients | | **-0.38** | -0.65 | -0.99 | **-0.34** | -0.48 | -0.44 | **-0.33** | **-0.34** | **-0.33** |
| p value | | **0.004** | 0.058 | 0.078 | **0.017** | 0.192 | 0.560 | **0.024** | **0.017** | **0.024** |
| Number of Observations | | 57 | 9 | 3 | 48 | 9 | 4 | 48 | 48 | 47 |
| **Nausea and Vomiting** | |  |  |  |  |  |  |  |  |  |
| Pearson Correlation Coefficients | | **-0.42** | -0.64 | **-1.00** | -0.26 | -0.37 | -0.97 | -0.06 | -0.07 | -0.12 |
| p value | | **0.001** | 0.061 | **0.0049** | 0.077 | 0.362 | 0.169 | 0.709 | 0.653 | 0.411 |
| Number of Observations | | 57 | 9 | 3 | 47 | 8 | 3 | 47 | 47 | 46 |
| **Gas and Bloating** | |  |  |  |  |  |  |  |  |  |
| Pearson Correlation Coefficients | | **-0.29** | -0.35 | -0.99 | **-0.43** | -0.59 | -0.46 | -0.02 | -0.07 | -0.17 |
| p value | | **0.032** | 0.359 | 0.110 | **0.002** | 0.095 | 0.536 | 0.879 | 0.613 | 0.241 |
| Number of Observations | | 57 | 9 | 3 | 48 | 9 | 4 | 48 | 48 | 47 |
| **Constipation** | |  |  |  |  |  |  |  |  |  |
| Pearson Correlation Coefficients | | -0.27 | -0.61 | -1.00 | -0.27 | -0.46 | -0.60 | -0.27 | -0.28 | **-0.33** |
| p value | | 0.043 | 0.079 | 0.0417 | 0.063 | 0.210 | 0.402 | 0.067 | 0.056 | **0.024** |
| Number of Observations | | 57 | 9 | 3 | 48 | 9 | 4 | 48 | 48 | 47 |
| **Blood in Poop** | |  |  |  |  |  |  |  |  |  |
| Pearson Correlation Coefficients | | -0.23 | **-0.77** | -0.94 | -0.24 | -0.48 | **-1.00** | -0.01 | -0.11 | -0.01 |
| p value | | 0.087 | **0.015** | 0.217 | 0.010 | 0.193 | **0.003** | 0.968 | 0.477 | 0.973 |
| Number of Observations | | 57 | 9 | 3 | 48 | 9 | 4 | 48 | 48 | 47 |
| **Diarrhea** | |  |  |  |  |  |  |  |  |  |
| Pearson Correlation Coefficients | | -0.15 | 0.43 | 0.49 | **-0.42** | -0.48 | 0.29 | **-0.33** | **-0.39** | **-0.37** |
| p value | | 0.26 | 0.250 | 0.672 | **0.003** | 0.195 | 0.712 | **0.020** | **0.007** | **0.011** |
| Number of Observations | | 57 | 9 | 3 | 48 | 9 | 4 | 48 | 48 | 47 |
| **Worry About Going Poop** | |  |  |  |  |  |  |  |  |  |
| Pearson Correlation Coefficients | | -0.10 | -0.17 | 0.49 | -0.09 | -0.03 | 0.05 | -0.20 | -0.15 | -0.13 |
| p value | | 0.457 | 0.660 | 0.672 | 0.569 | 0.936 | 0.949 | 0.194 | 0.323 | 0.400 |
| Number of Observations | | 57 | 9 | 3 | 43 | 8 | 4 | 43 | 43 | 42 |
| **Worry About Stomach Aches** | |  |  |  |  |  |  |  |  |  |
| Pearson Correlation Coefficients | | -0.11 | -0.24 | **-1.00** | -0.17 | -0.46 | -0.12 | 0.13 | 0.12 | 0.02 |
| p value | | 0.433 | 0.541 | **0.0049** | 0.262 | 0.252 | 0.872 | 0.405 | 0.452 | 0.904 |
| Number of Observations | | 57 | 9 | 3 | 44 | 8 | 4 | 44 | 44 | 43 |
| **Medicines** | |  |  |  |  |  |  |  |  |  |
| Pearson Correlation Coefficients | | 0.03 | 0.05 | **-1.00** | -0.04 | 0.44 | -0.01 | 0.04 | -0.01 | -0.28 |
| p value | | 0.842 | 0.892 | **0.0049** | 0.8078 | 0.381 | 0.995 | 0.792 | 0.944 | 0.084 |
| Number of Observations | | 55 | 9 | 3 | 40 | 6 | 3 | 40 | 40 | 39 |
| **Communication** | |  |  |  |  |  |  |  |  |  |
| Pearson Correlation Coefficients | | -0.22 | **-0.35** | -0.81 | -0.22 | -0.29 | -0.99 | 0.11 | 0.08 | -0.26 |
| p value | | 0.097 | **0.007** | 0.402 | 0.174 | 0.527 | 0.079 | 0.480 | 0.639 | 0.100 |
| Number of Observations | | 56 | 57 | 3 | 41 | 7 | 3 | 41 | 41 | 40 |
| **GSM total score** | |  |  |  |  |  |  |  |  |  |
| Pearson Correlation Coefficients | | **-0.35** | -0.55 | -0.98 | **-0.41** | -0.61 | -0.59 | -0.20 | -0.24 | **-0.32** |
| p value | | **0.007** | 0.122 | 0.115 | **0.004** | 0.081 | 0.413 | 0.162 | 0.094 | **0.028** |
| Number of Observations | | 57 | 9 | 3 | 48 | 9 | 4 | 48 | 48 | 47 |
| **GI symptoms total score** | |  |  |  |  |  |  |  |  |  |
| Pearson Correlation Coefficients | | **-0.36** | -0.57 | -0.99 | **-0.43** | -0.63 | -0.53 | -0.23 | -0.27 | **-0.30** |
| p value | | **0.006** | 0.109 | 0.099 | **0.003** | 0.071 | 0.466 | 0.114 | 0.063 | **0.042** |
| Number of Observations | | 57 | 9 | 3 | 48 | 9 | 4 | 48 | 48 | 47 |
|  | Correlation: perfect +/-1, high degree between +/- 0.50 and +/-1, moderate between +/-0.30 and +/-0.49, low between +/- 0.29. p-value <0.05 considered significant and marked in bold text. GSM total score- 74 items, GI symptoms total score- 58 items. | | | | | | | | | |

Table S6. Positive Pearson correlations for clinical factors and scales.

|  | Parent report  clinical factors | | Child report  clinical factors | |
| --- | --- | --- | --- | --- |
| **Scale** | “Wound infection” | “Ischemic bowel” | “Wound infection” | “Ischemic bowel” |
| Trouble Swallowing |  |  |  |  |
| Pearson Correlation Coefficients | **0.34** | **0.34** | **0.45** | **0.45** |
| p value | **0.010** | **0.010** | **0.001** | **0.001** |
| Number of Observations | 57 | 57 | 48 | 48 |
| Constipation |  |  |  |  |
| Pearson Correlation Coefficients | 0.17 | 0.17 | **0.29** | **0.29** |
| p value | 0.199 | 0.199 | **0.042** | **0.042** |
| Number of Observations | 57 | 57 | 48 | 48 |
| Worry About Going Poop |  |  |  |  |
| Pearson Correlation Coefficients | **0.39** | **0.39** | **0.51** | **0.51** |
| p value | **0.002** | **0.002** | **0.0005** | **0.0005** |
| Number of Observations | 57 | 57 | 43 | 43 |
| Communication |  |  |  |  |
| Pearson Correlation Coefficients | **0.28** | **0.28** | 0.27 | 0.07 |
| p value | **0.034** | **0.034** | 0.093 | 0.674 |
| Number of Observations | 56 | 56 | 41 | 41 |
| The variables were coded as follows, wound infection (yes=1, no=2) and ischemic bowel (yes=1, no=2) Quality of Life scores ranges from 0 (worst) to 100 (best). This means that the Pearson Correlation Coefficients reflect that absence of wound infection or ischemic bowel is correlated with better quality of life. Correlation: perfect +/-1, high degree between +/- 0.50 and +/-1, moderate between +/-0.30 and +/-0.49, low between +/- 0.29. p-value <0.05 considered significant, marked in bold text. | | | | |

Table S7 PedsQL gastrointestinal symptoms scales and worry scales means for GS parent report compared to Esophageal atresia/ Hirschsprung’s disease/ Functional constipation.

| **Variable** | **Gastroschisis (n=58)** | **Esophageal atresia (n=32)** | **p-value** | **Hirschsprungs disease (n=32)** | **p-value** | **Functional constipation (n=38)** | **p-value** |
| --- | --- | --- | --- | --- | --- | --- | --- |
| **Stomach Pain and Hurt** | 81.4 (19.0) | 84.0 (16.3) | 0.52 | 70.1 (18.0) | **0.0075** | 70.1 (20.1) | **0.007** |
| **Stomach Discomfort When Eating** | 87.5 (18.2) | 90.9 (13.5) | 0.35 | 78.8 (18.8) | 0.035 | 78.5 (15.8) | **0.017** |
| **Food and Drink Limits** | 90.9 (19.6) | 83.5 (18.1) | 0.080 | 69.3 (30.6) | **0.0008** | 78.6 (24.2) | **0.0082** |
| **Trouble Swallowing** | 98.5 (3.9) | 77.1 (24.1) | **<.0001** | 94.5 (11.9) | 0.073 | 95.3 (10.9) | 0.086 |
| **Heart Burn and Reflux** | 92.7 (10.5) | 85.5 (16.9) | **0.037** | 87.5 (14.1) | **0.053** | 86.5 (14.2) | **0.026** |
| **Nausea and Vomiting** | 95.1 (10.7) | 93.4 (12.1) | 0.51 | 90.2 (15.5) | 0.13 | 85.8 (18.9) | **0.0084** |
| **Gas and Bloating** | 77.0 (23.4) | 85.5 (14.5) | **0.039** | 49.2 (22.0) | **<0.0001** | 63.4 (26.6) | **0.010** |
| **Constipation** | 82.3 (19.1) | 81.8 (16.9) | 0.91 | 66.1 (16.9) | **0.0002** | 61.7 (26.9) | **0.0002** |
| **Blood in Poop** | 95.6 (10.4) | 96.5 (10.2) | 0.70 | 95.7 (12.1) | 0.97 | 90.1 (18.6) | 0.11 |
| **Diarrhea** | 92.3 (12.4) | 91.9 (13.5) | 0.88 | 68.9 (20.1) | **<0.0001** | 85.7 (14.1) | **0.017** |
| **Worry About Going Poop** | 94.6 (10.8) | 93.1 (12.0) | 0.54 | 77.0 (21.1) | **<0.0001** | 81.7 (17.3) | **0.0001** |
| **Worry About Stomach Aches** | 88.8 (21.0) | 92.6 (17.3) | 0.39 | 80.1 (29.1) | 0.14 | 73.7 (28.3) | **0.0064** |
| **Medicines** | 95.3 (13.0) | 86.5 (17.9) | **0.020** | 86.4 (23.0) | 0.061 | 77.8 (25.8) | **0.0003** |
| **Communication** | 79.6 (27.0) | 71.8 (30.3) | 0.22 | 61.8 (31.8) | **0.0069** | 67.7 (30.1) | **0.049** |
| **GSM total score** | 87.5 (11.7) | 85.8 (10.4) | 0.49 | 72.6 (13.5) | **<0.0001** | 75.3 (14.0) | **<0.0001** |
| **GI symptoms total score** | 87.0 (12.7) | 86.1 (10.5) | 0.72 | 71.9 (13.5) | **<0.0001** | 75.3 (14.7) | **<0.0001** |
| For comparison between groups t-test was used for continuous variables. For continuous variables Mean (SD) is presented. When variances are not equal(p<0.05) the SD is based on Satterthwaite´s approximation, otherwise  the SD is based on the pooled SDs. P value represents comparison between Gastroschisis - Esophageal atresia, Gastroschisis- Hirschsprung’s Disease, Gastroschisis-Functional Constipation  GSM total score- 74 items, GI symptoms total score- 58 items. The response options of the PedsQL Gastro-intestinal scales were transformed to a linear score scale with 0 denoting worst and 100 best quality of life. p< 0.05 marked in bold text, considered significant. | | | | | | | |

Table S8. PedsQL gastrointestinal symptoms scales and worry scales means for child report compared to Esophageal atresia/ Hirschsprung’s disease/ Functional constipation.

| **Variable** | **Gastroschisis (n=58)** | **Esophageal atresia (n=20)** | **p-value** | **Hirschsprung´s disease (n=26)** | **p-value** | **Functional constipation (n=26)** | **p-value** |
| --- | --- | --- | --- | --- | --- | --- | --- |
| **Stomach Pain and Hurt** | 77.6 (21.4) | 77.5 (16.5) | 0.99 | 78.8 (17.6) | 0.80 | 70.5 (20.0) | 0.17 |
| **Stomach Discomfort When Eating** | 88.3 (16.1) | 87.0 (16.9) | 0.77 | 88.4 (16.0) | 0.98 | 83.1 (16.3) | 0.19 |
| **Food and Drink Limits** | 86.9 (24.7) | 88.7 (13.7) | 0.69 | 84.1 (22.3) | 0.64 | 77.6 (26.7) | 0.14 |
| **Trouble Swallowing** | 92.9 (14.2) | 79.2 (21.7) | **0.015** | 97.8 (7.3) | 0.058 | 93.3 (8.5) | 0.89 |
| **Heart Burn and Reflux** | 86.4 (15.7) | 86.3 (12.8) | 0.96 | 87.7 (10.5) | 0.67 | 86.1 (13.4) | 0.92 |
| **Nausea and Vomiting** | 93.8 (12.9) | 92.5 (10.8) | 0.71 | 88.7 (16.7) | 0.16 | 90.1 (12.5) | 0.25 |
| **Gas and Bloating** | 76.3 (23.0) | 81.9 (17.8) | 0.34 | 65.0 (23.9) | **0.051** | 67.3 (28.0) | 0.14 |
| **Constipation** | 78.0 (19.5) | 82.2 (16.3) | 0.39 | 75.1 (15.5) | 0.53 | 64.3 (24.6) | **0.011** |
| **Blood in Poop** | 95.2 (14.4) | 100.0 (0.0) | **0.027** | 98.1 (7.7) | 0.27 | 92.3 (14.6) | 0.41 |
| **Diarrhea** | 89.7 (14.5) | 89.1 (12.7) | 0.88 | 75.6 (19.3) | **0.0007** | 82.0 (20.7) | 0.10 |
| **Worry About Going Poop** | 91.4 (17.4) | 93.8 (10.6) | 0.51 | 84.4 (15.8) | 0.10 | 84.6 (15.4) | 0.11 |
| **Worry About Stomach Aches** | 85.8 (25.5) | 88.8 (16.2) | 0.58 | 80.3 (29.6) | 0.42 | 74.5 (23.8) | 0.074 |
| **Medicines** | 90.9 (19.2) | 85.6 (21.2) | 0.34 | 78.5 (22.3) | **0.020** | 74.3 (30.4) | **0.017** |
| **Communication** | 81.6 (28.5) | 73.9 (25.6) | 0.31 | 74.4 (25.3) | 0.30 | 65.5 (25.4) | **0.022** |
| **GSM total score** | 84.6 (11.9) | 85.1 (10.3) | 0.87 | 80.0 (10.6) | 0.10 | 75.9 (14.6) | **0.0073** |
| **GI symptoms total score** | 84.0 (12.3) | 85.1 (10.2) | 0.73 | 80.2 (10.8) | 0.19 | 76.2 (15.7) | **0.022** |
| For comparison between groups t-test was used for continuous variables. For continuous variables Mean (SD) is presented. When variances are not equal(p<0.05) the SD is based on Satterthwaite´s approximation, otherwise  the SD is based on the pooled SDs. P value represents comparison between Gastroschisis - Esophageal atresia, Gastroschisis- Hirschsprung’s Disease, Gastroschisis-Functional Constipation. GSM total score- 74 items, GI symptoms total score- 58 items. The response options of the PedsQL Gastro-intestinal scales were transformed to a linear score scale with 0 denoting worst and 100 best quality of life. p< 0.05 marked in bold text, considered significant. | | | | | | | |
